# Retraction: MicroRNA-182 downregulates Wnt/β-catenin signaling, inhibits proliferation, and promotes apoptosis in human osteosarcoma cells by targeting HOXA9

**DOI:** 10.18632/oncotarget.28269

**Published:** 2022-09-06

**Authors:** Zi-Feng Zhang, Yong-Jian Wang, Shao-Hua Fan, Shi-Xin Du, Xue-Dong Li, Dong-Mei Wu, Jun Lu, Yuan-Lin Zheng

**Affiliations:** ^1^Key Laboratory for Biotechnology on Medicinal Plants of Jiangsu Province, School of Life Science, Jiangsu Normal University, Xuzhou 221116, P.R. China; ^2^Department of Orthopedics, The Third Affiliated Hospital, Shenzhen University, Shenzhen 518002, P.R. China; ^*^Co-first authors

**This article has been retracted:** In July of 2020, the Academic Committee of Jiangsu Normal University launched an investigation of this paper and requested that the major authors provide the original experimental records and data for verification. To date, however, the authors have failed to provide the requested documents. The committee has concluded that this paper violates academic integrity. In light of this, the paper is retracted.

Original article: Oncotarget. 2017; 8:101345–101361. 101345-101361. https://doi.org/10.18632/oncotarget.21167

